# Magnesia

**Published:** 1845-04

**Authors:** 


*Magnesia.* — M. Mialhe, in the "Journal de Pharmacie," June, 1844, has a paper on the different varieties of magnesia, which presents some points worthy of attention. Calcined magnesia is imported into France from this country, and the French practitioners find a very marked difference in the therapeutic effects of the imported and homemade magnesia, for which M. Mialhe undertakes to account. He says that the calcined magnesia which comes from London has been exposed, after calcination, either intentionally or accidentally, to a moist atmosphere, and has absorbed water to the extent of from 12 to 20 per cent. This he regards as an imperfect hydrate of magnesia. When the French calcined magnesia is made into a draught, it solidifies into a paste; the English remains permanently fluid. There is between the two magnesias the same difference as exists between lime spontaneously slaked in the air, and lime suddenly slaked by the affusion of water. The latter only is capable of forming a solid mortar, and is therefore exclusively employed in masonry.

There is a very close analogy between lime and magnesia; there is a caustic or quick magnesia, as there is a caustic or quick lime; and there is a slaked magnesia, as there is a slaked lime.

Anhydrous or caustic magnesia has very little affinity for carbonic acid; consequently, when calcined magnesia is kept in a dry place, it will remain for years without combining with the carbonic acid of the atmosphere; and even when it has attracted water, and become converted into hydrate, it has far less affinity for carbonic acid than is generally supposed. It is almost insoluble in acids; but it is in its caustic or anhydrous state that magnesia must be employed to convert balsam of copaiba into pills.

Ignorance of this point has caused several samples of copaiba to be rejected as bad, because hydrated magnesia was employed to solidify it, which it does not effect.

Caustic magnesia should never be employed in medicine, at least in large doses; first, because it is more difficult of solution in the acids of the stomach than hydrated magnesia; second, because its property of combining with water, and rendering solid ten times its own weight, would, if given in large doses, be likely seriously to injure the coats of the stomach. Hence the thirst which follows the administration of calcined magnesia, and the tenesmus from conglomeration in the lower bowels. These symptoms often oblige patients to discontinue the use of magnesia.

M. Mialhe suggests that practitioners should be careful not to employ freshly prepared calcined magnesia, or rather, that in order to have a preparation of uniform strength, calcined magnesia should be combined with one-fourth part of its weight of water, which he proposes to call magnesia hydrated to one-fifth, and he says this forms by far the best form in which magnesia can be employed medicinally.—*Ib.*

